# Basal Cell Carcinomas in Gorlin Syndrome: A Review of 202 Patients

**DOI:** 10.1155/2011/217378

**Published:** 2010-09-28

**Authors:** Elizabeth A. Jones, Mohammed Imran Sajid, Andrew Shenton, D. Gareth Evans

**Affiliations:** ^1^Genetic Medicine, Manchester Academic Health Science Centre, University of Manchester and St Mary's Hospital, Central Manchester University Hospitals NHS Foundation Trust, Oxford Road, Manchester M13 9WL, UK; ^2^University of Manchester, Oxford Road, Manchester M13 9PL, UK

## Abstract

Gorlin syndrome (Naevoid Basal Cell Carcinoma Syndrome) is a rare autosomal dominant syndrome caused by mutations in the *PTCH* gene with a birth incidence of approximately 1 in 19,000. Patients develop multiple basal cell carcinomas of the skin frequently in early life and also have a predisposition to additional malignancies such as medulloblastoma. Gorlin Syndrome patients also have developmental defects such as bifid ribs and other complications such as jaw keratocysts. We studied the incidence and frequency of basal cell carcinomas in 202 Gorlin syndrome patients from 62 families and compared this to their gender and mutation type. Our data suggests that the incidence of basal cell carcinomas is equal between males and females and the mutation type cannot be used to predict disease burden.

## 1. Introduction

Gorlin syndrome (also known as Naevoid Basal Cell Carcinoma Syndrome and Basal Cell Naevus Syndrome) is characterised by the presence of a variety of developmental anomalies and predisposition to a range of cancers. A major and problematic feature of the disorder is the development of multiple basal cell carcinomas (BCCs) which begin to appear from the early teens. Other features of Gorlin Syndrome include a recognizable facial appearance with milia, macrocephaly, bossing of the forehead, hypertelorism, and coarse facial features which is present in 60% of individuals with Gorlin Syndrome [1–3]. Examination of the palms reveals the presence of pits in the palm of the hand in most affected individuals, which are characteristic. Jaw keratocysts are common and often multiple and tend to begin in the second decade of life. Skeletal anomalies are also common and include bifid ribs, wedge-shaped vertebrae, and a short 4th metacarpal. Ectopic calcification, particularly in the falx, is present in more than 90% of affected individuals by age 20 years [3]. For reviews see [1, 4].

In 1993 Evans et al. devised clinical criteria for making a diagnosis of Gorlin Syndrome based on the most frequent and/or specific features of the syndrome [3]. These have since been modified in an attempt to improve diagnostic accuracy [2, 4].  Table 1 details current diagnostic criteria and a diagnosis can be made when 2 major or 1 major and 2 minor criteria are fulfilled. 

Gorlin Syndrome is an autosomal dominant disorder with an estimated birth incidence of 1 in 19,000 individuals [5]. Penetrance is near complete [6] but there is extremely variable expressivity. The development of BCCs is one of the most problematic features of this disorder. The initial site of appearance of BCCs is most frequently the face and nape of the neck. The sites frequently involved are the face, back, and chest and they are rarely found below waist. The median age of onset is roughly 25 years but they may appear as early as two years of age or as late as 65 years of age (authors personal experience). BCCs can vary in number from only some to thousands. There is a significant tendency for proliferation between puberty and 35 years. BCCs in this syndrome behave in the same manner as sporadic BCCs and rarely metastasize. The histology of the BCC is also similar to that of sporadic BCCs and consists of nests and islands or sheets of large, deeply stained nuclei with indistinct cell membranes [7].

The numbers of BCCs that develop have shown a significant association with skin pigmentation and sun exposure; only 40% of African-American Gorlin syndrome patients present BCCs with the number of lesions normally less compared to the many BCCs in Caucasians. Shanley et al. (1994) compared the phenotype of Australian Gorlin Syndrome patients with those from the English survey by Evans et al. [3] and found that multiple BCCs present from an earlier age in the Australian population and postulated that this maybe a sign of greater exposure to ultraviolet (UV) radiation [8]. Radiation therapy triggers proliferation in the BCCs [2] and can cause huge numbers to occur particularly after radiotherapy in childhood [9]. 

Gorlin syndrome results from mutations in the *PTCH* gene, the human homolog of the *Drosophila* segment polarity gene ***patched*** (***ptc***) [10, 11]. It is interesting to note that 50% of sporadic BCCs have been found to have mutations in this gene [12]. *PTCH* encodes a transmembrane glycoprotein (made up of 1447 amino acids) with 12 transmembrane domains and two extracellular loops, and a putative sterol sensing domain. Alternative splicing results in multiple transcript variants encoding different isoforms. *PTCH* is an integral component of the hedgehog signalling pathway, which serves many developmental and regulatory roles. 

Analysis of *PTCH* mutations in Gorlin syndrome patients has identified deletions, insertions, splice site alterations, nonsense, and missense mutations. There does not appear to be a particular hot spot for mutations [13]. It is not known if the mutation type correlates with the number of BCCs that develop. Approximately 20%–30% of probands have a *de novo* mutation in *PTCH,* and some of these will be somatic mosaics. Mosaicism is caused when a mutation arises early in development. The resulting individual will be a mixture of cells, some cells with the mutation and some cells without the mutation. How early in development the mutation occurs will determine what tissue(s) and what percentage of cells will have the mutation. If the mutation is not present in a significant proportion of blood cells, then the mutation will not be detectable by routine mutation analysis.

The development of associated malignancies in Gorlin Syndrome is thought to arise from the classic two-hit suppressor gene model: baseline heterozygosity secondary to germline *PTCH* mutation as the first hit, with the second hit due to mutagen exposure such as UV or ionizing radiation. Loss of heterozygosity seems to also be the mechanism for jaw cysts whereas congenital malformations may be attributable to alterations in the concentration of the* PTCH* gene product in the dosage sensitive hedgehog signalling pathway [14].

We have assessed the presence, onset, and number of BCCs in individuals with Gorlin syndrome and identified whether presence or type of PTCH mutation or gender impacts on this.

## 2. Methods

Identified cases of Gorlin Syndrome in the North West Region were collected from the regional Gorlin Syndrome Register (Genetic Medicine, St Mary's Hospital, Manchester), which was established in 1990 by DGE [3] as the result of a study begun in 1982 by Dr P Farndon. All individuals on the register have been found to have a mutation in the PTCH gene or meet clinical criteria for a diagnosis of Gorlin Syndrome. In the current study, 202 patients from 62 families were ascertained from the Gorlin Syndrome register. The clinical details for each patient were recorded from the hospital notes. SPSS and MedCalc were used for data analysis, and Kaplan-Meier curves were drawn. Mutation types were defined as (1) truncating mutation (small frameshift deletions or insertions or nonsense mutations), (2) splice site donor or acceptor; (3) missense mutations; (4) large single or multiexon deletions.

## 3. Results

There were 202 confirmed cases of Gorlin Syndrome on the register database, which included 100 females and 102 males. All bar one individual/family was of white European origin with the only exception of Indo-Asian descent. 158 patients had been tested for mutations in PTCH, and mutations have been identified in 96 (61%). Untested affected family members were assumed to have the same result as a tested proband. BCCs had been diagnosed in 135/202 (67%) of Gorlin Syndrome patients. Similar proportions of males (64%) and females (70%) had been diagnosed with a BCC. 67 individuals had not had a BCC. Only four individuals with Gorlin Syndrome and aged greater than 50 years had no BCCs, and the majority without BCCs were <40 years of age (44/67) with nearly half (30/67) being <20 years of age. Five individuals with *PTCH* mutations did not have a diagnosed BCC after 40 years of age. 

**Figure 1:**
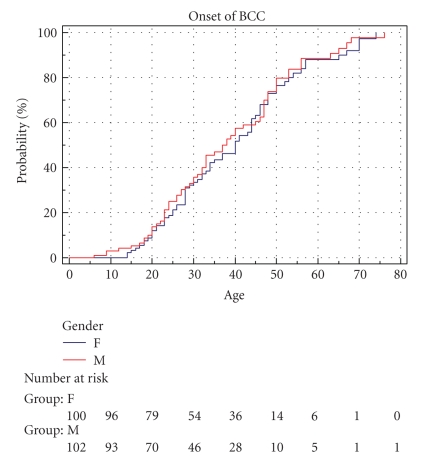
Cumulative onset of BCC by gender. Male(m) *n* = 100. Female (f) *n* = 102.

We first studied the incidence of BCCs in males and females in our cohort.  Figure 1 shows Kaplan-Meier curves of cumulative incidence of BCCs with age comparing male patients and female patients. Both curves rise in a similar manner and are close together. The curves (and data) show that the cumulative incidence of BCCs at age 20 years in male patients is 13.7% and 12% in female patients. By 50 years of age, 76.5% of females and 80% of males had been diagnosed with a BCC. A long rank test was performed assuming similarity in the curves, and this produced a *P*-value of  .625 with a Hazard ratio of 0.92 for females against males. These results show that male Gorlin patients do not have a higher cumulative incidence of BCCs with age than female patients. 

**Figure 2:**
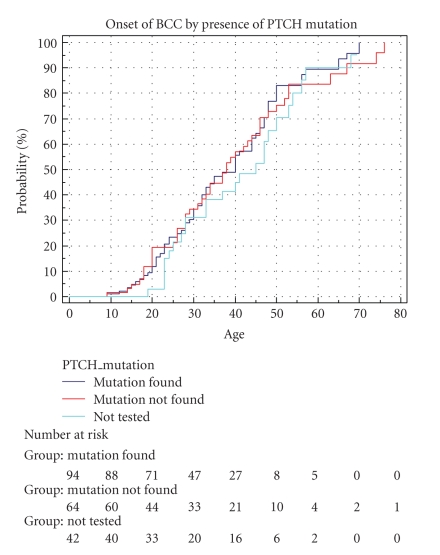
Cumulative onset of BCC by presence of *PTCH* mutation. Mutation found (y) *n* = 94. Not found (n) *n* = 64. Untested (u) *n* = 42.

We next divided our cohort of patients into 3 groups: those with a known mutation, those in whom no mutation had been identified, and those in whom the mutation status was unknown.  Figure 2 shows Kaplan-Meier curves of cumulative incidence of BCCs with age comparing patients with (1) patients with an identified *PTCH* mutation; (2) patients in whom the mutation status is unknown; (3) patients in whom no mutation was found. All three curves rise in a similar manner and are again very closely aligned. The curves (and data) show that the cumulative incidence of BCCs at age 20 years in patients with a mutation is 12%, 3% in those with an unknown status, and 12% in those in whom a mutation was not found. At 30 years the cumulative incidence of BCCs in patients with a mutation is 34.5%, 32% in those with an unknown status, and about 34.5% in those in whom a mutation was not found. A long rank test was performed assuming similarity in the curves, and this produced a *P*-value of  .616. These results show that patients with a *PTCH* mutation (i.e., those with definite Gorlin syndrome) do not have a higher cumulative incidence of BCCs with age than those in whom a mutation was not found and in those whom the mutation status is unknown. Therefore, it does not appear that individuals who may be somatic mosaics for a *PTCH* mutation but fulfill diagnostic criteria or whose Gorlin syndrome could be caused by an as yet unidentified gene have a lower BCC load.

**Figure 3:**
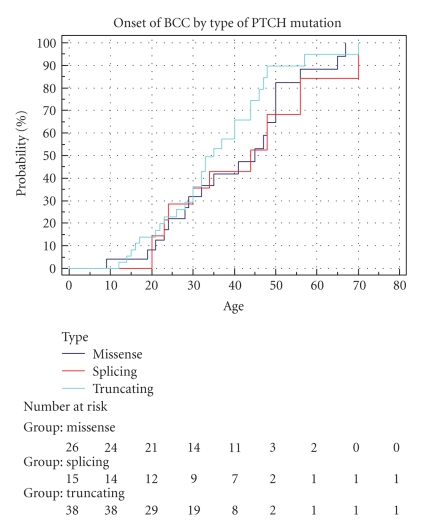
Cumulative onset of BCCs by type of *PTCH* mutation. Missense (mis) *n* = 26. Splicing (spl) *n* = 15. Truncating (tru) *n* = 38.

**Figure 4:**
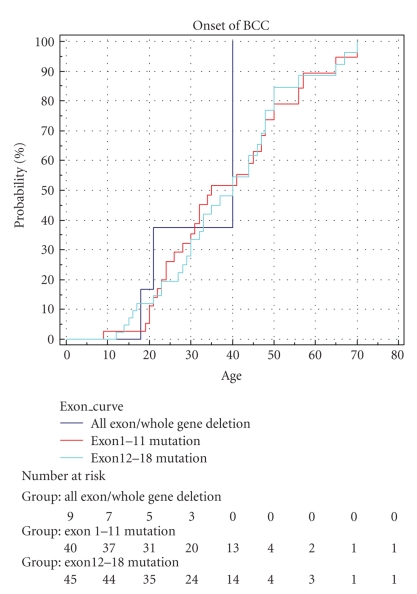
Effect of mutation position on onset of BCCs.

We next assessed if type of mutation affected incidence of BCC. Ninety-four individuals with identified mutations were assessable for BCC incidence (truncating mutation *n* = 38, splice-site mutation *n* = 15, large single or multiexon deletion *n* = 15, and missense mutation *n* = 26).  Figure 3 shows Kaplan-Meier curves of cumulative incidence of BCCs with age comparing patients with three mutation types. All three curves rise in a similar manner and are closely aligned; the large deletion curve is shown as part of Figure 4. The curves (and data) show that the cumulative incidence of BCCs at age 30 years in patients with a truncating mutation is 29%, 29% in those with a splice-site mutation, and 32% in those with a missense mutation. A long rank test was performed assuming similarity in the curves, and this produced a *P*-value of  .38. These results show that patients with a truncating mutation do not have a higher cumulative incidence of BCCs with age than those with a splice-site mutation or missense mutation. This suggests that missense mutations are not associated with a milder form of Gorlin syndrome than truncating or splice-site mutations.

Finally, the effect of mutation position was assessed. The *PTCH* gene was divided into two portions, exons 1-11 and exons 12-18. Whole gene deletions were treated as a separate group. Figure 4 again shows no significant difference in BCC onset dependant on mutation position (*P* = .617).

## 4. Discussion

Gorlin syndrome is characterized by the presence of multiple BCCs, yet little is known about whether gender or mutation type influences the age at onset and number of tumours that develop. Gorlin syndrome is known to be the result of mutations in the *PTCH* gene. The mutation detection rate is low at 61% in the Manchester series but this is similar to most other reports in Gorlin Syndrome [15–18]. Some of this can be explained by somatic mosaics in who the mutation is not detectable in blood. Mutation analysis has not been carried out in a systematic way, and this may also contribute to the low detection rate. 

To date, no firm evidence for a genotype/phenotype correlation in Gorlin syndrome has been demonstrated, and there is adequate variation in single families to believe that environmental exposure and perhaps modifier genes may justify much of the variation. We have shown that presence or absence of a *PTCH* mutation, mutation type, and gender have virtually no impact on the age at which the first BCCs develop or the numbers of BCCs that develop. This is clinically relevant as the finding of a specific *PTCH* mutation does not appear to give prognostic information about the likely age of onset of BCC or the clinical disease burden. 

Mutation position and type of mutation have been shown to affect phenotype for a number of tumour-prone disorders including neurofibromatosis type 2 (NF2) [19, 20] and familial adenomatous polyposis [21]. Although the numbers with detected mutations are relatively small, they should be large enough to detect a potential hypomorphic effect of missense mutations in *PTCH* in our study or a severe effect of truncating mutations as seen in NF2.

It is interesting to note that biallelic loss of *PTCH* in adult conditional *PTCH*-knockout mice developed tumours of the skin on the tails and ears. These tumours had features of human nodular BCCs. The inactivation of *PTCH* at different timepoints in this mouse model predisposed to the development of different tumour types. For example, rhabdomyosarcoma results from the prenatal inactivation of *PTCH* whereas BCC do not. This study demonstrates that the time point and mode of inactivation of the second allele affect the spectrum of associated tumours and may explain some of the clinical variation seen in this disorder [22].

A major problem for Gorlin patients is the sheer number of BCCs which can develop. Only a small fraction of these become invasive but they can invade deep underlying structures, especially in the face. Different treatment modalities are used in these patients: surgical excision, Mohs micrographic surgery, cryotherapy, photodynamic therapy, ablative laser therapy, and topical 5% imiquimod [4]. Trials are also underway investigating the role of specific hedgehog pathway inhibitors in treatment. It will be interesting to see if mutation positive patients respond differently to these agents than mutation negative patients.

## 5. Conclusion

In Gorlin Syndrome patients, neither the age of onset of BCCs or the numbers of BCCs that develop can be predicted by the presence or absence of a *PTCH* mutation or by mutation type. Males and females appear to be equally affected by BCCs.

## Figures and Tables

**Table 1:** Diagnostic criteria for Gorlin Syndrome. A diagnosis can be made when 2 major or 1 major and 2 minor criteria are fulfilled.

Major criteria	Minor criteria
Lamellar (sheet-like) calcification of the falx or clear evidence of calcification in an individual younger than age of 20 years	Childhood medulloblastoma
Jaw keratocyst	Lympho-mesenteric or pleural cysts
2 or more palmar/plantar pits	Macrocephaly (OFC >97th centile)
Multiple BCCs (more than five in a lifetime) or a BCC before age of 30 years.	Cleft lip/palate
First degree relative with Gorlin Syndrome	Vertebral/rib anomalies such as bifid/splayed/extra ribs or bifid vertebrae
	Preaxial or postaxial polydactyly
	Ovarian/cardiac fibromas
	Ocular anomalies (cataract, developmental defects, and pigmentary changes of the retinal epithelium)
